# Gut microbial composition varies with host metabolic phenotype in juvenile Atlantic salmon

**DOI:** 10.1242/jeb.251523

**Published:** 2026-05-14

**Authors:** Elle C. Lindsay, Alexandru S. Barcan, Neil B. Metcalfe, Abby L. E. Bryce, Philip McGinnity, Martin S. Llewellyn, Bachar A. Cheaib

**Affiliations:** ^1^ School of Biodiversity, One Health and Veterinary Medicine, Graham Kerr Building, University of Glasgow, Glasgow G12 8QQ, UK; ^2^ Institute for Ocean Engineering, Shenzhen International Graduate School, Tsinghua University, 518055 Shenzhen, China; ^3^ School of Biological, Earth & Environmental Sciences, University College Cork, Cork, Ireland, T23 TK30; ^4^ Department of Infectious Diseases, division of Microbiology and Hygiene, Medical Faculty, Heidelberg University, Im Neuenheimer Feld 324, Heidelberg D-69120, Germany

**Keywords:** *Salmo salar*, Gut microbiota, Standard metabolic rate, Growth efficiency, Rhodobacteraceae

## Abstract

Standard metabolic rate (SMR) influences growth, behaviour and energy use in fish, yet its relationship with gut microbiota remains unclear. Here, we combined physiological measurements with 16S rRNA sequencing of foregut and hindgut tissue to test whether gut microbial communities differ with metabolic phenotype in juvenile Atlantic salmon. High-SMR fish showed greater growth efficiency and lower body water content than low-SMR fish, indicating higher fat levels. In contrast, microbial differences were most evident in the foregut, where low-SMR fish exhibited significantly higher alpha diversity. Microbial beta-diversity analyses revealed clear segregation among metabolic groups, and distance-based redundancy analysis showed that both SMR and body mass strongly explained variation in foregut microbial composition. Correlation analysis identified a negative association between SMR and members of the Rhodobacteraceae family, which were consistently more abundant in the foregut of low-SMR fish. Together, these findings indicate that the metabolic phenotype is associated with distinct patterns of energy utilisation and gut microbiota composition, suggesting that foregut microbial communities may contribute to individual differences in metabolic strategy.

## Teaser

**Summary:** Metabolic phenotype is associated with differences in growth efficiency and foregut microbiota in juvenile Atlantic salmon, linking energy allocation to distinct microbial community patterns.

## INTRODUCTION

Metabolic rate underpins key biological traits such as growth, reproduction and survival (Chabot et al., 2016; White and Kearney, 2013; White and Seymour, 2004; Artacho and Nespolo, 2009). Variations occur between and within species, influenced by factors such as activity level, temperature and body size (Auer et al., 2016). Standardised measurements such as basal metabolic rate (BMR) in endotherms and standard metabolic rate (SMR) in ectotherms facilitate comparisons (White and Kearney, 2013), yet significant interspecific and intraspecific variation persists (Pettersen et al., 2018). While metabolism has a genetic component (White and Kearney, 2013), phenotypic variation persists as a result of environmental effects (Robertson et al., 2014). In salmonids, body composition (fat and water content) fluctuates markedly with seasonal changes in food availability and temperature. These shifts determine overwinter survival, growth potential and major life-history transitions such as maturation and migration (Berg and Bremset, 1998; Elliott, 1976a; Swift, 1955; Iosilevskii et al., 2022). Because metabolic rate influences both energetic demand and the accumulation of fat reserves, variation in SMR may contribute to individual differences in energy allocation. This salmonid-specific energetic context provides biological motivation for examining links between SMR, growth efficiency, fat storage and gut microbiota. Metabolic rate also influences food processing (Millidine et al., 2009) and behaviour. For example, higher aerobic scope in Atlantic cod (*Gadus morhua*) allows longer foraging in hypoxic environments (Behrens et al., 2018).

Metabolic rate correlates with angling vulnerability in largemouth bass (*Micropterus salmoides*) (Redpath et al., 2010), dominance and aggression in Atlantic salmon (*Salmo salar*) (Metcalfe et al., 1995; Cutts et al., 1998), and risk taking in common carp (*Cyprinus carpio*) (Huntingford et al., 2010). While a high SMR may enhance competitive ability (Metcalfe et al., 1995), it can be disadvantageous during food scarcity because of increased energy demands (Cutts et al., 2002). Elevated SMR may necessitate dominance to secure necessary resources (Cutts et al., 2002). Juvenile Atlantic salmon with higher SMR process food more rapidly, allowing faster nutrient utilisation, but incur higher metabolic costs during digestion, affecting assimilation efficiency and suggesting higher growth potential (Millidine et al., 2009). An additional factor that may contribute to unexplained metabolic variation is the gut microbiota, which plays key roles in nutrient absorption, energy recovery and overall metabolic regulation (Ayayee et al., 2020; Cani and Delzenne, 2009). Given these behavioural and physiological consequences of metabolic variation, understanding how such traits connect with gut microbiota provides an important next step. Emerging research highlights complex interactions between gut microbiota and host physiology that influence metabolic rate (Ayayee et al., 2020; Cani and Delzenne, 2009). Gut microbiota adapt to changes in host diet (Bergmann et al., 2015; Li et al., 2024; Corbin et al., 2023), altering microbial metabolites that, in turn, influence host metabolism (Kovatcheva-Datchary et al., 2015; Ruiz et al., 2024; Kokou et al., 2022). These metabolites can signal to peripheral organs, thereby regulating host metabolism (Schroeder and Bäckhed, 2016). The composition of the gut microbiota is highly context dependent, responding to ontogeny and host developmental stage (Burns et al., 2016), environmental factors (Rudi et al., 2018; Candela et al., 2012), and metabolic states including hibernation (Carey et al., 2013).

The benefits of specific metabolic traits are context dependent (Archer et al., 2021), explaining the persistence of phenotypic diversity (Burton et al., 2011). For instance, juvenile Atlantic salmon with high routine metabolic rates perform well in stable habitats but lose this advantage in harsher conditions (Reid et al., 2012). Although controlled laboratory studies indicate that higher SMR is linked to faster growth, investigations of wild brown trout (*Salmo trutta*) populations show either an absence of this pattern or a reversed, negative association (Álvarez and Nicieza, 2005). In Atlantic salmon, the correlation between metabolic rate and survival varies across environments, suggesting environmental heterogeneity maintains variation in metabolic phenotypes (Robertson et al., 2014). Environmental factors, including food availability (Auer et al., 2015a), temperature (Khaliq and Hof, 2018) and hypoxia (Jordan and Steffensen, 2007), influence the intraspecific metabolic rate variation.

Salmonids experience pronounced fluctuations in energy demand driven by changes in food availability, temperature and environmental conditions (Birnie-Gauvin et al., 2021). Flexibility in metabolic rate and gut microbiota composition allows fish to adapt to diverse environments (Zhang et al., 2025). Examining both aspects could reveal relationships between metabolism, gut microbiota and performance. Fish can reduce metabolic costs during periods of low food availability, although this capacity varies among individuals (O'Connor et al., 2000). Changes in food input alter metabolic rate (Auer et al., 2015a) and gut microbiota composition (Green et al., 2013; Heikkinen et al., 2006), with consequences for overall fitness. Because gut microbiota contribute to digestion (Nayak, 2010), and may influence growth, a relationship between metabolic phenotype, the gut microbiome and growth efficiency in teleosts exists. In this study, we define ‘growth efficiency’ as the proportion of ingested energy converted into somatic growth, ‘calorie recovery’ as the fraction of dietary energy retained after accounting for faecal losses, and ‘energy allocation’ as the partitioning of energy between maintenance and growth. To our knowledge, no previous study has directly explored whether SMR is associated with gut microbiota composition in a teleost fish.

Research across multiple fish species shows that gut microbiota can influence digestion, nutrient absorption and energy use, suggesting a potential link between microbial community composition and metabolic phenotype (Butt and Volkoff, 2019; Lindsay et al., 2020; Sullam et al., 2012). The gut microbiota respond dynamically to variation in diet, feeding frequency, temperature and environmental conditions (Wu et al., 2025) and these shifts can alter the production of microbial metabolites that affect host physiology and energy balance (Yukgehnaish et al., 2020).

Because salmonids experience substantial fluctuations in food availability and energetic demand, changes in gut microbial composition may contribute to individual differences in growth, fat storage and energy recovery (Fraser et al., 1995; Finstad et al., 2011). This potential interaction between metabolic rate and gut microbiota highlights an important gap in our understanding of metabolic variation in fish. Because the foregut (pyloric caecum) is the primary site of nutrient uptake in salmonids, microbial differences in this region may be especially relevant to metabolic variation (Zarkasi et al., 2016; Ringø et al., 1995).

The objective of this study was twofold: to assess whether variation in SMR influences physiological performance and to test whether gut microbiota contribute to differences in energy consumption. We first quantified energy allocation in juvenile Atlantic salmon with contrasting SMR by assessing growth efficiency and fat storage under a controlled environment. We then characterised gut microbiota composition to determine whether microbial diversity or structure is associated with metabolic phenotype. Based on previous work linking microbial diversity with digestive efficiency (Kovatcheva-Datchary et al., 2015; Nayak, 2010), we hypothesised that juvenile Atlantic salmon with lower SMR would exhibit higher gut microbial diversity and greater energy recovery efficiency than fish with higher SMR.

## MATERIALS AND METHODS

### Fish husbandry and acclimation

The Atlantic salmon, *Salmo salar* Linnaeus 1758, used in this experiment were of wild origin, derived from parents caught during their spawning migration in the River Conon, Northern Scotland, as part of hydropower mitigation measures. The fish, part of a larger experiment described by Auer et al. (2018), were reared under hatchery conditions from the egg stage at the aquarium facilities of the School of Biodiversity, One Health and Veterinary Medicine, University of Glasgow. At the start of the experiment in July 2017, all fish were immature juveniles in their second summer of the parr stage. Atlantic salmon typically spend 1–3 years in freshwater before transitioning to the smolt stage and migrating to the ocean (Rinaldo et al., 2024). Sixty fish were randomly selected from a 400 l circular stock tank containing several hundred offspring from 30 full-sibling families (see Auer et al., 2018, for details). These fish were transferred into individual compartments (190×130×200 mm) within a recirculating stream system in the same aquarium room. All shared a common water source sterilised by a UV filter. The room was maintained on a 12 h:12 h light:dark photoperiod, and the water temperature was kept at 11.9±1.0°C, similar to the stock tank.

The fish acclimated in these compartments for 2 weeks, during which each was hand-fed a daily ration of EWOS MICRO 5P LR pellets (EWOS Ltd, Bathgate, UK). The ration was determined by weighing five randomly selected stock fish to estimate the mean (±s.d.) mass (15.8±4.5 g) of the experimental fish. This mean mass was used to calculate the daily ration for all experimental fish during acclimation, based on an equation from Auer et al. (2015b), itself derived from Elliott (1976b), which describes the energetics and growth of brown trout; the ration (in MJ day^−1^) was midway between maintenance and maximum intake:
(1)

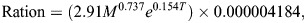
where *M* is body mass (g) and *T* is water temperature (°C).

Using the feed's energetic content (19.53 MJ kg^−1^), the daily ration (mg pellets) for the 60 fish was calculated based on the average body mass and water temperature during acclimation. The energetic value of the pellets was provided by the manufacturer (EWOS Ltd). Two individuals died during the acclimation period of unknown causes, leaving a sample size of 58 fish.

This study was approved by the School of Veterinary Medicine Research Ethics Committee, University of Glasgow.

### Establishment of experimental groups

To accurately measure SMR in fish, individuals must be thermally acclimated, in a post-absorptive state and as inactive as possible (Chabot et al., 2016). Although respirometry is often used, opercular ventilation rate (VR) correlates closely with metabolic rate (oxygen consumption) in juvenile salmon (Millidine et al., 2008) This non-invasive method minimises disturbance and was used to distinguish between fish with ‘low’ or ‘high’ SMR.

After 2 weeks of acclimation, experimental groups were established. First, each fish's SMR was estimated by recording its VR while undisturbed in individual tanks after a 48 h fasting period. VR was recorded for 20 s (expressed as beats min^−1^) and repeated three times for each fish, with measurements taken an hour apart. The mean VR was calculated from these measurements. All observations occurred during the light period on the same day, with minimal disturbance to the aquarium.

Fish have to be inactive and undisturbed in order to guarantee accurate SMR measurement. Dissolved oxygen was monitored throughout recordings and remained above 90% saturation in all tanks. Therefore, the fish rested on the substrate because the water flow was sufficient for turnover but slow enough to prevent the need for active swimming. After acclimation and the feeding regime, VR was measured only when fish were resting calmly and showed no overt behavioural response to disturbance. VR measurements below 30 beats min^−1^ were excluded from the mean calculation, as such low rates (analogous to bradycardia) indicate a brief stress response and do not represent true SMR (Wendelaar Bonga, 1997; Barton, 2002). Accepted mean VR measurements ranged from 34 to 101 beats min^−1^. In order to correct SMR for body mass, fish were measured immediately after the final VR recording. Each was anaesthetised using benzocaine solution, weighed to the nearest 0.1 g, and measured (fork length) to the nearest 0.1 mm. Three individuals with visible fungal infections, all from the same shared aquarium system, were removed from the experiment to avoid potential confounding of microbiota, resulting in a final sample of 55 fish (mean±s.d. mass 13.9±3.9 g, length 107.1±10.5 mm).

Regression equations from Millidine et al. (2008) were then used to relate VR to SMR for the remaining 55 individuals, with knowledge of fish mass (*M*, in g) and water temperature (*T*, in °C):
(2)

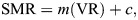
where *m* is the slope of the VR–SMR relationship, calculated as:
(3)

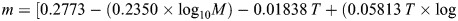
*c* is the intercept of the VR–SMR relationship, calculated as:
(4)

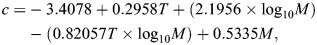
and VR is expressed as beats min^−1^. Note that the value for *m* includes a correction for an error in the published equation. All equations employed in this study, together with definitions of variables and their specific roles, are summarised in Table S1.

Fish that had not been feeding or producing faeces during acclimation were excluded from further analysis. The estimated SMR values (mg O_2_ h^−1^) were corrected for body mass, as it influences metabolic and growth rates (Auer et al., 2015b). SMR was plotted against mass (*M*, g), and the resulting regression (SMR=0.3544×*M−*2.4856, *R*^2^=0.70) generated the expected SMR for each fish based on its mass. The expected SMR was subtracted from the actual SMR to obtain the relative SMR (rSMR). Positive residuals indicate a higher SMR than expected for their mass, while negative residuals indicate a lower SMR. Fish were ranked based on their rSMR, and the 15 individuals with the highest and lowest rSMR values were selected for further research. This resulted in two groups (*n*=15 per group) with distinct metabolic phenotypes: high SMR and low SMR.

### Feeding regime and growth measurements

Growth efficiency, defined as the ratio of mass gain to the amount of food consumed, reflects how effectively fish convert feed into body mass (Seyedalmoosavi et al., 2022). Each fish was fed an ‘intermediate’ ration, which was smaller than an *ad libitum* amount but expected to be fully consumed. Unique rations were calculated for each fish using Eqn 1 above, except that the value for an individual fish's wet mass used in Eqn 1 was in this instance estimated from the equation linking fish fork length *L* (mm) to mass *M* (g) for the experimental population of fish:
(5)

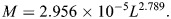
This gave a predicted mass for each fish, based on its length.

The reason for using the predicted mass rather than the measured mass was that one aim of Eqn 1 is to correct ration size for fish size, and using length gave a more accurate representation of the fish's size, uninfluenced by its current body condition. The energy value (MJ day^−1^) of the daily ration from Eqn 1, along with the energy content (19.53 MJ kg^−1^) of the feed, determined the mass of feed (mg) to be fed daily to each fish. Each individual received its ration in one meal. Before feeding, any uneaten food from the previous day was noted and removed by siphoning. Knowledge of food consumption, fish body mass, body length and energy content was necessary for subsequent growth efficiency calculations. To calculate the changes in energy content of the fish during the experiment, the initial energy density (kJ) of each individual was estimated using equations derived from Elliott (1976a):
(6)

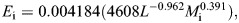
where *E*_i_ is the initial energy density (kJ g^−1^, wet mass), *L* is fork length (cm) and *M*_i_ is the initial wet mass (g); the multiplier 0.004184 converts the calories in the original equation to kJ. The value given by Eqn 5 was multiplied by the wet mass of the fish to give an estimate of its total energy content at the beginning of the experiment (*G*_i_, kJ). At the end of the experimental period, the final energy density (*E*_f_, kJ g^−1^) of each fish was estimated using a further equation from Elliott (1976a):
(7)

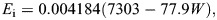
where *W* is the percentage water content.

In order to determine *W*, fish were euthanised via benzocaine overdose followed by spinal severing. Wet mass (to the nearest 0.01 g) and fork length (to the nearest 0.1 mm) were recorded (mean±s.d. mass 15.43±4.82 g, length 113.9±11.3 mm). In total, 55 fish were included in the final dataset after removing individuals that died or showed signs of fungal infection. Each fish contributed two gut samples, one from the foregut and one from the hindgut, resulting in 110 gut samples, which were stored at −20°C for subsequent microbiome analysis. All dissections were performed using sterile instruments under aseptic conditions. The external surface of the fish was rinsed with 70% ethanol, and the body cavity was opened using ethanol-flamed tools. The pyloric caeca (foregut) and distal intestine (hindgut) were removed with sterile forceps and placed into sterile microcentrifuge tubes. Instruments were re-sterilised with ethanol and flame after each fish to prevent cross-contamination.

To calculate water content, we recorded the wet mass of each fish after gut removal (hereafter referred to as *M*_f_; mean±s.d. 14.38±4.34 g, range 7.93–24.47 g). This value was used in water content and energy density calculations and reflects the carcass mass excluding gastrointestinal tissue. Each carcass was partitioned into three pieces and dried at 60°C for approximately 70 h. Dry mass was then recorded (mean±s.d. 3.73±1.20 g, range 1.93–6.19 g), allowing calculation of percentage water content (mean±s.d. 73.65±2.26%, range 67.09–75.61%). One fish from the high metabolic rate group was excluded because of data entry error. The percentage water content was also used as proxy for fat content, as there is a strong negative correlation between water and fat content (*r*=−0.98); fat contains less water than muscle because protein binds water whereas fat does not (Elliott, 1976a).

The wet mass energy density (kJ g^−1^) was calculated using Eqn 6 above. This value was then multiplied by each fish's final wet mass after gut removal (*M*_f_) to determine total carcass energy content (*G*_f_, in kJ). Energy gained by the fish during the experiment (*E*_gain_, kJ) was calculated by subtracting *G*_i_ from *G*_f_. This value was scaled using equations by Elliott and Hurley (2000) to give the energy gained by a fish of a standardised size of 10 g (*E*_corr_, kJ) in order to make the data comparable for fish of different size:
(8)

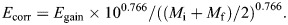
Finally, growth efficiency was calculated by dividing *E*_corr_ by the energy consumed by each fish (also standardised to a 10 g fish using a variant of Eqn 8 with energy consumed replacing *E*_gain_). Growth efficiency ranged from 0 to 1.0, where 1.0 indicates complete conversion of ingested energy into new body energy content.

For growth efficiency analysis, only data from fish that consumed their full ration each day for over 90% of the experimental period were used, ensuring accurate total energy consumption values. This resulted in growth efficiency data for 18 fish (10 from the high SMR group and 8 from the low SMR group).

### Environmental and Atlantic salmon parr samples: collection and processing

#### Atlantic salmon faeces for bomb calorimetry

Faecal samples were collected over 10 days and aggregated for each individual in order to assess the energy content of each fish's faeces using bomb calorimetry and so compute the energy obtained from the diet. Each day, all faecal material produced in the preceding 24 h was siphoned from the tank, provided the fish had consumed its full ration the previous day to ensure quantifiable energy intake. Samples were stored at −20°C in 1 l containers and added to daily.

After 10 days, faecal samples and residual water were defrosted and centrifuged at 168 ***g*** for 5 min in 50 ml tubes to remove excess water. This process was repeated until all samples were centrifuged, producing a single pellet per individual, representing up to 10 days of collection. Pellets were stored at −20°C for subsequent analysis.

The energy content of each individual's faeces was determined by bomb calorimetry, yielding the energy content (kJ g^−1^) of the collected faecal material. The energy content of the food pellets was 19.53 kJ g^−1^ (as provided by the manufacturer). By accounting for the mass of food consumed and the energy remaining in faeces, the nutritional energy each fish gained from its feed was calculated. Data were collected for 23 of the 30 fish; the remaining seven had insufficient faecal mass for bomb calorimetry.

The daily relative energy retained (DRER) (kJ) of each individual was defined as:
(9)

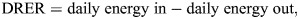
where daily energy in and daily energy out (kJ) were calculated as:
(10)

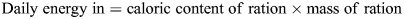
and
(11)

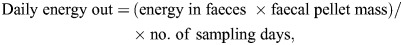
where caloric content and energy are in kJ g^−1^, and mass is in g. The term ‘relative’ is used because the absolute energy retained cannot be precisely calculated; the ‘daily energy out’ value was derived using wet faecal mass, which included some water content. As the dry mass of the faeces was unknown, it was assumed all faecal samples had equivalent water content.

#### Atlantic salmon faeces for microbial load analysis

Additional faecal samples were collected for DNA extraction and quantitative PCR (qPCR) analysis on days separate from those used for DRER measurements. Faeces from each tank were collected using a pipette on two separate days, providing duplicate samples for each fish. Samples were stored in 15 ml centrifuge tubes, centrifuged at 4500 rpm for 5 min to remove excess water, and the resulting pellets were stored at −80°C for later analysis.

DNA extraction was performed using the QIAamp DNA Stool Mini Kit (Qiagen) with modifications to the manufacturer's protocol. Only dissected gut tissue was used for 16S rRNA sequencing, whereas faecal samples were used exclusively for qPCR-based microbial load estimation and bomb calorimetry. Buffer ASL (Qiagen) was added directly to the frozen faecal samples in the centrifuge tubes, with the volume adjusted relative to the sample mass. Tubes were thoroughly vortexed before incubation. Lysis temperature was increased to 90°C for 30 min to enhance the breakdown of hard-to-lyse bacteria. Following the protocol, DNA was quantified using NanoDrop spectrometry.

qPCR was employed to assess bacterial load by measuring fluorescence relative to the presence of target DNA, using general bacterial primers amplifying the 16S rRNA gene. The primers used in the qPCR reactions were U16SRT-F, 5′-ACTCCTACGGGAGGCAGCAGT-3′ and U16SRT-R, 5′-TATTACCGCGGCTGCTGGC-3′. This primer set, taken from Clifford et al. (2012), was designed to amplify products from bacterial 16S rRNA genes without the need for a probe, by aligning >960,000 bacterial 16S rRNA sequences. Amplification and detection of DNA by qPCR were performed with the MX3000P qPCR System (Agilent Technologies). The output, the CT value, represents the cycle at which fluorescence surpasses a threshold and is proportional to the logarithm of bacterial quantity (Nadkarni et al., 2002). Furthermore, to create a standard curve for qPCR, competent *E. coli* from a StrataClone PCR Cloning Kit (Agilent Technologies) were grown overnight in Luria–Bertani (LB) broth at 37°C. Two cultures were prepared: LBB1 (30 ml LB broth+1 ml inoculum) and LBB5 (30 ml LB broth+5 ml inoculum). Optical density measurements were taken at various time points over 5 h to gauge growth rates from different starting concentrations. Viable cell counts were determined by plating serial dilutions (10° to 10^−11^, made with PBS) of each culture on LB agar plates. Each dilution was plated in duplicate, with 6–8 drops (20 μl each) per plate. Plates were incubated at 37°C for 12 h, and colonies (colony-forming units, CFU) were counted from the 10^−5^ dilution plate.

DNA was isolated from the *E. coli* culture using the QIAamp DNA Stool Mini Kit (Qiagen), omitting the InhibitEX tablet step. Extracted DNA was quantified with a Qubit fluorometer. Combining CFU data with DNA concentration determined that the inoculum contained 117,897.7 CFU ng^−1^ DNA. Serial dilutions of the extracted *E. coli* DNA (1, 1/2, 1/4, 1/8 and 1/16, in triplicate) were quantified using qPCR to create a standard curve for bacterial quantification. This curve was used to quantify faecal samples from the experimental fish. Thirty faecal samples were quantified from the first sampling session and 27 from the second, as three fish did not produce faeces on the second occasion. CFU data were log-transformed to normalise distributions, and average values were calculated from duplicate measurements for each individual.

All samples were run in duplicate for the determination of DNA by qPCR. The reaction was performed using a total volume of 20 μl: 10 μl SensiMix™ SYBR No-ROX Master Mix (Bioline), 1 μl of each the forward and reverse primers, 3 μl water and 5 μl of template DNA per well. The reaction conditions were as follows: 95°C for 10 min; 40 cycles of 95°C for 15 s, 58°C for 15 s and 72°C for 15 s; followed by a single cycle of 95°C for 1 min, 58°C for 30 s and 95°C for 30 s. For subsequent data analysis, the MxPro qPCR software was used.

#### Environmental samples

Throughout the experimental period, environmental samples were collected every 4 days to assess and control for the background microbial diversity in the tank environment. Biofilm samples were taken by swabbing the inside of two randomly selected stream tank compartments. Water samples were collected by passing 1 l of water from the stream tank through a Minisart single-use filter (16534-K, CE 0120) using a peristaltic pump. Biofilm and water microbiota were analysed to characterise the environmental microbial background but were not included as predictors in the statistical models. Instead, they served as contextual controls to confirm that gut communities were distinct from the surrounding environment. Each filter paper was manually removed from the filter and immediately placed into a cryotube (Cryo-Vial Int Thd FS, Ref: LW3534) before being stored in liquid nitrogen for subsequent analysis. These environmental samples were taken in triplicate and processed for DNA extraction, PCR and sequencing alongside tissue samples.

#### Environmental and Atlantic salmon gastrointestinal tissue samples for microbial analysis

**Fig. 1.**
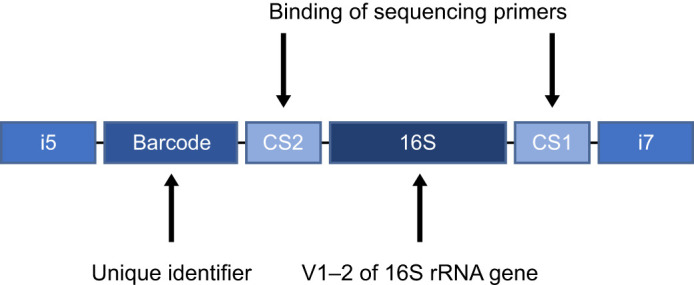
**The 16S rRNA gene construct generated after primary and second-round PCR.** Amplification of variable region (V)1–2 of the 16S rRNA gene was achieved, alongside addition of CS1 and CS2 tags, in primary PCR. A DNA barcode for identification and Illumina index sequences i5 and i7 were added during second-round PCR. This product was then sequenced.

For DNA extraction from tissue (foregut and hindgut) and environmental samples, the QIAamp DNA Stool Mini Kit (Qiagen) was used with modifications to the manufacturer's protocol. Buffer ASL (Qiagen) was added directly to the frozen tissue samples, which were then transferred to 2.0 ml microcentrifuge tubes (Thermo Scientific #3469-11) containing a 1/4-inch (6.35 mm) ceramic bead and lysing matrix A garnet (MP Biomedicals). The tubes were homogenised using a FastPrep machine at speed 4 for four rounds of 25 s prior to the lysis step. DNA concentration of environmental and tissue samples was quantified using NanoDrop spectrometry following the manufacturer's protocol. For primary PCR reactions targeting variable regions V1–V2 of the 16S rRNA gene, the primer pair CS1_27F and CS2_338R was used, adapted from previous studies on Atlantic salmon gut microbiota (Gajardo et al., 2016). The forward primer sequence was 5′-AGAGTTTGATCMTGGCTCAG-3′ and the reverse primer was 5′-GCTGCCTCCCGTAGGAGT-3′, targeting the V1–V2 region of the 16S rRNA gene. CS1 and CS2 tags were added to the 5′ ends of these primers for subsequent barcoding (see Fig. 1). To minimise amplification bias, PCR reactions were performed in triplicate and pooled after amplification. The V1–V2 region was selected because previous work on salmonids demonstrated that it minimises cross-amplification of salmon mitochondrial 16S sequences, which commonly occurs when using V3–V4 or V4–V5 primer sets (Heys et al., 2020; Werner et al., 2012).

Each 30 μl PCR reaction contained 1.5 μl of each primer (10 μmol l^−1^), 15 μl of Q5 Hot Start High-Fidelity 2× Master Mix (New England BioLabs Inc.) and 2 μl of DNA template. The PCR conditions were: initial denaturation at 95°C for 10 min; 30 cycles of 30 s at 95°C, 30 s at 55°C and 30 s at 72°C; followed by a final extension at 72°C for 10 min. PCR products were verified on a 1.7% agarose gel using TBE buffer.

These PCR products served as templates for a second-round PCR to attach DNA barcodes for sequencing and sample demultiplexing. A universal forward primer PE1_CS1_Fwr (5′-AATGATACGGCGACCACCGAGATCTACAC TGACGACATGGTTCTA-3′) and a barcoded reverse primer specific to each sample (5′-CAAGCAGAAGACGGCATACGAGATXXXXXXXTACGGTAGCAGAGACTTGGTCT-3′) were used, where ‘XXXXXXX’ represents the unique barcode sequence (Fig. 1). Each 25 μl reaction contained 12.5 μl Q5 Hot Start High-Fidelity 2× Master Mix, 1 μl forward primer, 1 μl barcode primer and 8 μl of DNA template. PCR conditions were: initial denaturation at 95°C for 10 min; 8 cycles of 10 s at 95°C, 30 s at 60°C and 1 min at 72°C; followed by a final extension at 72°C for 3 min. Barcoded PCR products were verified on a 1.7% agarose gel.

If multiple bands were observed upon gel visualisation, the desired band was excised and purified using the PureLink Quick Gel Extraction Kit (Invitrogen). Single-band products were purified using Agencourt AMPure XP beads (Beckman Coulter) with a modified 0.8:1 bead-to-product volume ratio. DNA concentrations were determined using a Qubit fluorometer. Amplicons were pooled in equal concentrations, and the final library was sequenced using the Illumina MiSeq system at Glasgow Polyomics, University of Glasgow.

### Data analysis

#### Statistical analysis

All statistical analyses were performed in R.3.5.1 using moments (https://CRAN.R-project.org/package=moments), rms (https://CRAN.R-project.org/package=rms) and e1071 (https://CRAN.R-project.org/package=e1071) packages for model diagnostics. Prior to analysis with models, all continuous variables were mean-centred and scaled to reduce the risk of multicollinearity, tested for using the rms package in R (https://CRAN.R-project.org/package=rms). Generalised linear models (GLMs) and linear models (LMs) were used to investigate potential relationships among microbial load, metabolism, growth efficiency and nutritional energy harvest. Because growth efficiency was calculated for only 18 out of the 30 Atlantic salmon, parallel models were run including and excluding growth efficiency as an explanatory variable. This approach assessed the effects of growth efficiency while retaining statistical power when it was not significant. Non-significant terms were removed, and final models were selected based on Akaike's information criterion corrected (AICc) and visual inspection of residual plots (scale-location, Cook's distance, *Q–Q* plots). Significance testing indicated the strength of observed relationships.

Graphs representing significant relationships from models with multiple explanatory variables were created by plotting the residuals of the response variable rather than the raw data. Plotting residuals enables visualisation of the effect of a focal predictor while statistically accounting for other variables in the model, offering a clearer interpretation than raw data when multiple covariates are present (Zuur et al., 2007).

#### Bioinformatic analysis

Quality assessment indicated that reverse reads were of higher quality than forward reads in MiSeq Illumina paired-end sequencing. Therefore, reverse reads were trimmed and filtered using Sickle (https://github.com/najoshi/sickle) with an average quality threshold above a Phred score of 30. Filtered sequences were decontaminated against the *S. salar* genome using DeconSeq (Schmieder and Edwards, 2011), chimeras were removed, and sequences were clustered at 97% similarity using VSEARCH (Rognes et al., 2016). First, *de novo* sequence clustering was performed with VSEARCH and then operational taxonomic units (OTUs) were taxonomically classified against the SILVA database (Quast et al., 2013) and annotated using the QIIME2 classifiers (Bolyen et al., 2019). OTUs were processed for multiple sequence alignment using MAFFT (Katoh and Standley, 2013) and an OTU tree was built using the software FASTTREE (Price et al., 2009) to assist with the calculation of beta diversity metrics (e.g. generalised UNIFRAC).

For downstream analysis, samples were separated by gut section [foregut (pyloric caecum) or hindgut] and analysed separately. A total of 27 foregut samples (13 from low SMR fish and 14 from high SMR fish) and 27 hindgut samples (14 low SMR and 13 high SMR) provided sufficient sequencing depth for analysis; some samples were excluded because of low sequencing quality.

Alpha diversity was assessed using the Shannon–Wiener index (*H*′). For improved interpretability, Shannon effective diversity [exp(*H*′)] was used in downstream analyses and reporting (Jost, 2006). The effective number of species expresses diversity as the equivalent number of equally abundant species, facilitating clearer biological interpretation. LMs were employed to explore relationships between microbial alpha diversity traits and variables such as metabolic rate (rSMR), percentage water content, fish mass, fish length, log average microbial load and experimental group (low or high SMR). In these models, microbial species richness or Shannon effective number was the response variable, and explanatory variables included experimental group, fish mass, percentage water content, DRER, rSMR and average microbial load. LMs also examined the effect of alpha diversity metrics on growth efficiency, with growth efficiency as the response variable and the aforementioned variables as explanatory factors. Non-significant terms were removed, and final models were selected based on AICc (using the MuMin package in R; https://CRAN.R-project.org/package=MuMIn) and visual inspection of residual plots (scale-location, Cook's distance, *Q–Q* plots). Significance testing was used to indicate the strength of observed relationships. As in previous analysis, when visualising alpha diversity data, residuals of the response variable were plotted instead of raw data to accurately represent relationships while controlling for other covariates in the model. Residual plotting isolates the relationship between the diversity metric and the predictor of interest while accounting for covariates.

Beta diversity was assessed using generalised UniFrac distance, generalised (Gunifrac) weighted and unweighted UniFrac methods (Chen et al., 2012). Visualisation of beta diversity was performed via unconstrained non-metric multi-dimensional scaling (NMDS), in which the phylogenetical distances of the microbial communities were assessed via PERMANOVA. Both alpha and beta diversity analysis were performed in R using the Rhea package (Lagkouvardos et al., 2017).

Taxonomic binning was performed using the SILVA database (Quast et al., 2013) to overview the genera present in each sample. Stacked bar plots displayed taxonomic composition and relative abundance in foregut and hindgut samples, with OTU abundance thresholds set at 0.25 or 0.5 for clarity. To identify microbial genera significantly differing in relative abundance between metabolic rate groups, the DESeqDataSetFromMatrix function from the DESeq2 package (Love et al., 2014) was used, with an adjusted *P*-value cut-off of 0.005 and log_2_ fold-change cut-off of 2. Negative binomial GLMs estimated OTU log-fold change between groups, Bayesian shrinkage provided shrunken log-fold changes, and the Wald test determined significance. Log_2_ fold-change was chosen for better data visualisation (Ijaz et al., 2018) and the Cox–Reid adjusted profile likelihood correction was used (Cox and Reid, 1987). Furthermore, to determine how explanatory variables contributed to variation in microbial communities, distance-based redundancy analysis (dbRDA) was used to examine the variation explained by environmental variables, incorporating the GuniFrac distance matrix (Shankar et al., 2017). Redundancy analysis with forward selection was performed to specifically select the environmental variables that explained variation within the microbial communities (Vass et al., 2020). Following forward selection with the ordistep function in the vegan package of R (https://CRAN.R-project.org/package=vegan), significant environmental variables were identified and a dbRDA was performed using the capscale command. Analysis was conducted separately for foregut and hindgut data. Statistical analysis of dbRDA results were performed using the adonis2 function, which employs PERMANOVA to test sample similarity based on GUniFrac distance.

Spearman correlation coefficients were calculated among metavariables (final wet mass, fish length, percentage water content, rSMR and average microbial load) and OTUs to assess correlations. A false discovery rate (FDR) correction was applied to adjust for type I errors. Correlation analysis was performed in R using the Rhea package (Lagkouvardos et al., 2017), with a *P*-value significance level of 0.05. To avoid underpowered analysis, OTUs present in less than 30% of samples were removed, and the minimum number of pairs required to support a correlation was set to four. Correlation results were visualised to determine whether the metabolic rate correlated with the presence of any OTUs.

## RESULTS

### Experimental group formation based on metabolic rate and morphology

To evaluate the impact of metabolic phenotype on energy allocation and growth efficiency in juvenile Atlantic salmon, fish were divided as described in Materials and Methods into two experimental groups based on their rSMR (Table 1). An unpaired two-sample *t*-test confirmed that there was no significant difference in mass between the high and low SMR fish groups (*t*_28_=1.05, *P*=0.30). This was expected because rSMR corrects SMR for body size; therefore, group differences reflect metabolic phenotype rather than differences in mass.

**Table 1.** Metabolic and body mass data for fish with high and low residual standard metabolic rate (SMR)

	High SMR group	Low SMR group
SMR (mg O_2_ h^−1^)	3.43±0.45	1.57±0.36
Mean rSMR	0.54	−0.70
rSMR range	−0.15 to 2.65	–1.35 to −0.38
Mass (g)	15.16±4.97	13.43±3.66
Mass range (g)	8.4 to 27.8	8.2 to 19.2

Metabolic and mass data for the 30 fish divided into two experimental groups based on residual SMR, corrected for fish mass. The groups consisted of 15 individuals with high SMR for their mass and 15 with low SMR, as determined by ventilation rate (VR) and subsequent prediction of SMR (mg O_2_ h^−1^). SMR values are presented as means±s.e.m. rSMR (relative SMR) data are means and range. Body mass is presented as mean±s.d. and range. No significant difference in body mass was observed between groups (*t*_28_=1.05, *P*=0.30), as expected when using rSMR, which accounts for body size.

### Larger juvenile Atlantic salmon retain more energy and produce lower-energy faeces

**Fig. 2.**
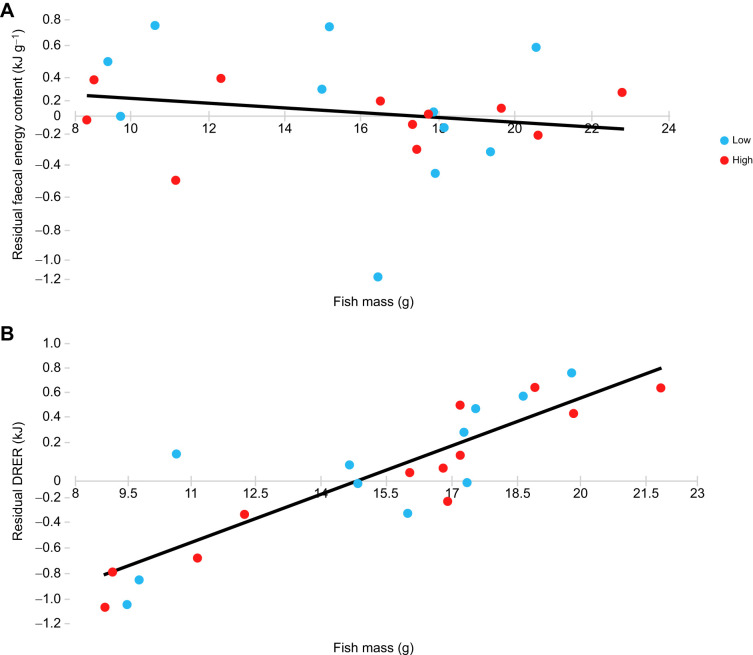
**Energy retention in juvenile Atlantic salmon.** (A) The relationship between residual faecal energy content and body mass as quantified by bomb calorimetry (*n*=22). Although statistical analysis was performed on absolute energy content, the data points plotted here are residual values after controlling for daily relative energy retained (DRER), log-transformed microbial load, percentage water content and relative standard metabolic rate (rSMR) so as to illustrate the relationship with body mass. (B) The relationship between residual daily DRER and body mass in juvenile Atlantic salmon (*n*=23). Data points are residual values after controlling for energy consumed. In A and B, blue and red points represent low and high SMR groups, respectively.

Faecal energy content (kJ g^−1^) was analysed using a LM, with fish mass, DRER, percentage water content, log-transformed average microbial load and rSMR as explanatory variables. The results indicated that faecal energy content was significantly related to fish mass (Fig. 2A; LM, *F*_5,16_=3.66, *P*=0.0105). Larger fish produced faeces with lower energy content per unit mass. Additionally, DRER increased with both fish mass (Fig. 2B; LM, *F*_2,20_=364.7, *P*<0.001) and the energy consumed (*P*<0.001). The method for calculating residual energy content and residual DRER can be found in the Supplementary Materials and Methods (see ‘Section 1: residual calculations for energy and DRER’).

### Growth efficiency correlates with increased rSMR and reduced water content

**Fig. 3.**
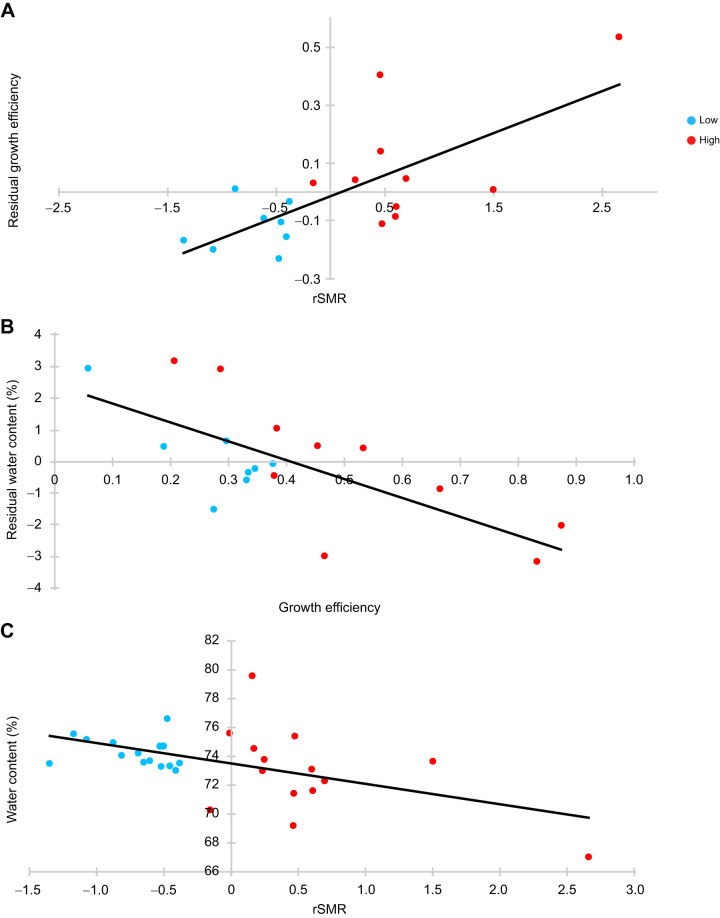
**Relationships between metabolic rate, growth efficiency and water content in juvenile Atlantic salmon.** (A) The relationship between residual growth efficiency and residual rSMR, with residual growth efficiency scaled to a 10 g fish (*n*=18). (B) The relationship between residual percentage water content and growth efficiency (standardised to a 10 g fish, *n*=18), after controlling for rSMR. (C) The relationship between percentage water content and rSMR (*n*=29). In all panels, blue and red points indicate individuals with low and high relative rSMR, respectively.

For growth efficiency analysis, only data from fish that consistently consumed their full ration for over 90% of the experimental period were considered, providing accurate measures of total energy intake. Growth efficiency (standardised to that of a 10 g fish) was found to increase with rSMR (Fig. 3A; LM, *F*_2,15_=10.26, *P*=0.0011) and fish mass (*P*=0.017), indicating that salmon with higher rSMR and larger mass were more efficient in converting food energy into growth. Details of the calculation for expected growth efficiency are provided in the Supplementary Materials and Methods (see ‘Section 2: growth efficiency and water content residuals’).

Notably, there was no significant relationship between total energy consumed and growth efficiency (LM, *F*_1,16_=0.57, *P*=0.46), nor between rSMR and total energy consumed (LM, *F*_1,28_=0.63, *P*=0.44), indicating that fish with higher rSMR were not consuming more food but were more efficient at utilising the energy they consumed. The percentage water content of the fish was used as an indicator of body composition. Water content (%) was negatively related to growth efficiency (Fig. 3B; LM, *F*_2,15_=44.94, *P*<0.001), suggesting that individuals that converted more energy into growth had lower water content, which is indicative of higher fat levels. For the subset of fish with growth efficiency data (*n*=18), there was no significant relationship between water content and rSMR (*P*=0.859). However, when data for all fish were included (*n*=29), a significant relationship emerged (Fig. 3C; LM, *F*_1,27_=9.94, *P*=0.0039), with higher SMR fish exhibiting lower water content, suggesting greater fat levels. Faecal samples were used only for physiological measurements (faecal energy content, DRER and microbial load). All microbiome sequencing was performed exclusively on foregut and hindgut tissue. Faecal material was not used for microbial community analysis.

### Foregut microbial diversity correlates with metabolic rate and growth efficiency in juvenile Atlantic salmon

**Fig. 4.**
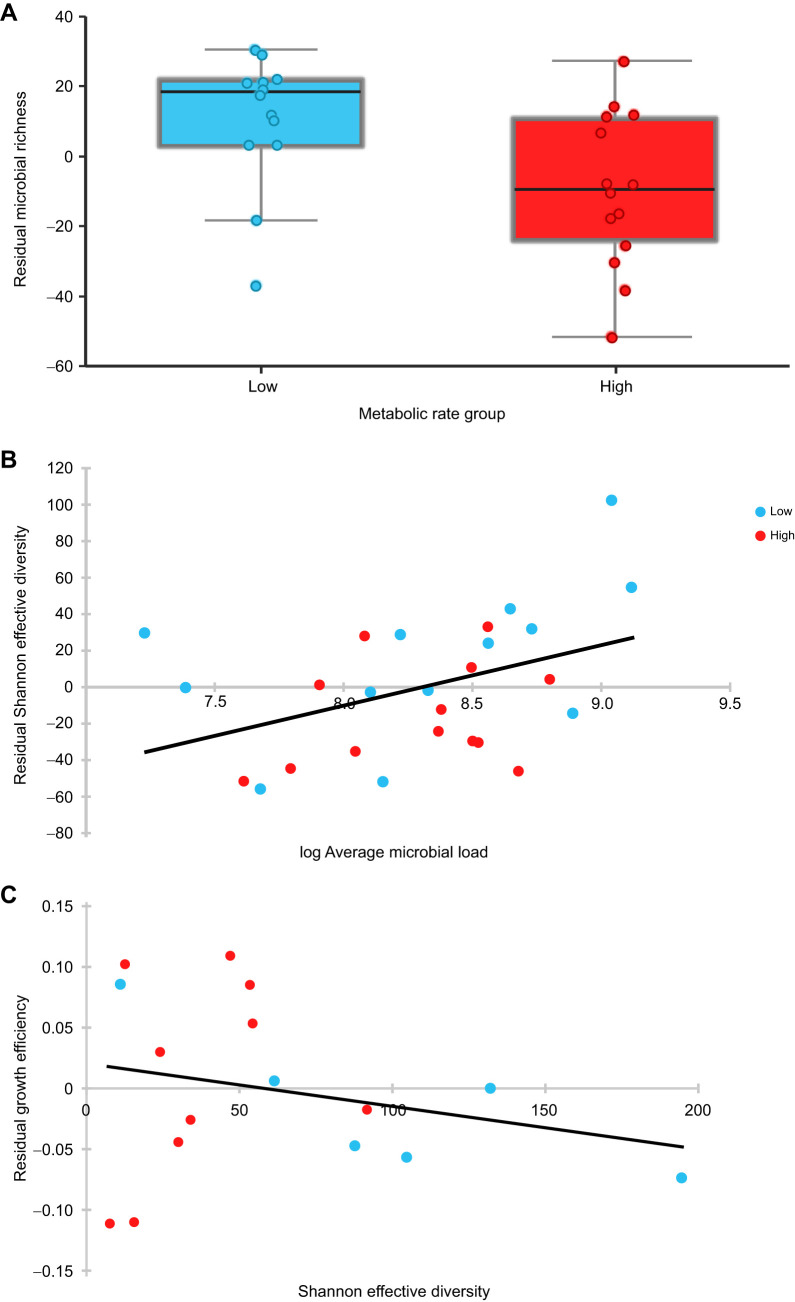
**Foregut microbial diversity in juvenile Atlantic salmon with low and high SMR.** (A) Difference in microbial richness between low and high SMR groups (*n*=27), with residual values plotted after controlling for microbial load. (B) Relationship between residual Shannon effective diversity and log-transformed microbial load (colony-forming units per gram, CFU g^−1^) in the foregut (*n*=26), controlling for water content. (C) Relationship between growth efficiency (standardised to 10 g fish mass) and Shannon effective diversity (*n*=16), controlling for microbial richness, microbial load and water content (*P*=0.043). Blue and red points represent low and high SMR groups, respectively. See Results for statistical analysis.

Analysis of the foregut microbiota revealed significant differences in alpha diversity between juvenile Atlantic salmon with low and high SMR. Fish with lower SMR exhibited higher microbial richness and Shannon effective diversity (Fig. 4A,B). Microbial richness differed significantly between the low and high SMR groups (LM, *F*_2,24_=5.05, *P*=0.023), and showed a positive trend with microbial load (colony-forming units per gram, CFU g^−1^; *P*=0.056). Shannon effective diversity also differed significantly between groups (LM, *F*_3,22_=7.17, *P*=0.019) and increased with microbial load (*P*=0.024) (Table 2).

**Table 2.** Linear model (LM) summary of microbial diversity and salmon physiology

Response	Explanatory	*t*-value	*P-*value
Richness	Experimental group – low SMR	2.43	0.023
	log-transformed average microbial load (CFU g^−1^)	2.01	0.056
Shannon effective	Experimental group – low SMR	2.54	0.019
	log-transformed average microbial load (CFU g^−1^)	2.42	0.024
	Fish water content (%)	1.46	0.16
Growth efficiency	Richness	2.93	0.014
	Shannon effective	−2.29	0.043
	log-transformed average microbial load (CFU g^−1^)	1.39	0.19
	Fish water content (%)	−8.22	<0.001

Summary of linear models testing the relationship between microbial alpha diversity (species richness or Shannon effective number) in the Atlantic salmon foregut and host physiological variables. The model formula was: Diversity∼Group+Mass+Water Content+DRER+rSMR+Microbial Load.

Growth efficiency (standardised to a 10 g fish mass) was positively associated with microbial richness (LM, *F*_4,11_=32.22, *P*=0.014) but negatively associated with Shannon effective diversity (*P*=0.043) and fish water content (*P*<0.001) (Fig. 4C, Table 2). These findings suggest that a higher number of OTUs in the foregut microbiota contributes to increased growth efficiency, whereas a more even distribution of OTU abundance (higher Shannon effective diversity) may be linked to decreased growth efficiency. Detailed statistical models, calculations of expected values and methodologies are provided in the Supplementary Materials and Methods [‘Section 3: microbial diversity metrics (foregut)’].

### Distinct foregut and hindgut microbiota profiles linked to low and high SMR

Microbial analyses were conducted on 18 fish that yielded complete sequencing data. This reduced sample size provides lower statistical power and may not detect smaller differences. To examine the difference in microbial beta diversity between the juvenile Atlantic salmon from the low and high SMR groups, multivariate analysis was used, which utilises generalised UniFrac metrics to account for the phylogenetic distance between OTUs.

**Fig. 5.**
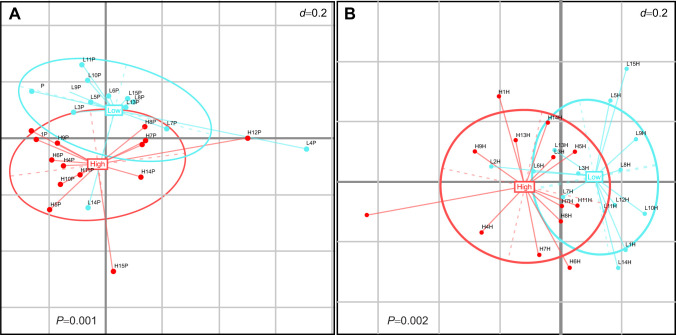
**The difference in gut beta diversity of juvenile Atlantic salmon with low or high SMR, illustrated by non-metric multidimensional scaling (NMDS) based upon generalised UniFrac.** Data are shown separately for the foregut (A) and hindgut (B). The dissimilarity scale of the grid, *d*=0.2, indicates that each grid line interval corresponds to approximately 20% dissimilarity between samples. The *P*-values were calculated by permutational multivariate analysis of variance, which used the distance matrix to assess whether the separation of groups (high and low SMR) was significant. Blue and red points represent fish with low and high metabolic rates, respectively.

The analysis of microbial beta diversity between low and high SMR groups showed significant differences in both the foregut and hindgut (Fig. 5).

**Fig. 6.**
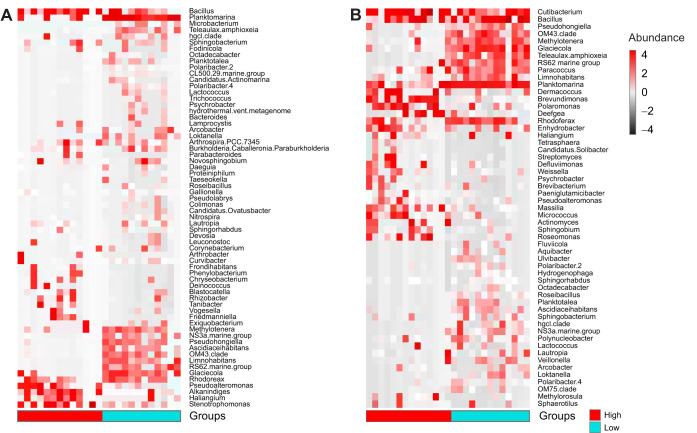
**Heatmaps showing microbial genera in the gut of juvenile Atlantic salmon classified as significantly different in abundance between low and high SMR groups.** Data are shown separately for the foregut (A) and hindgut (B). Genera were identified based on a log_2_-fold threshold. Each column represents a sample, colour coded by metabolic rate: blue for low SMR and red for high SMR. In the heatmaps, pink–red indicates increased abundance, while grey–black indicates decreased abundance. Differences in microbial composition between metabolic groups are displayed for both gut sections.

Further investigation into differentially abundant genera revealed 63 genera in the foregut and 55 in the hindgut that differed between low and high SMR groups (Fig. 6). In the foregut, 47 of the 63 differentially abundant genera were significantly more abundant in fish from the low SMR group (Fig. 6A). The most common differentially abundant genera belonged to Proteobacteria (e.g. *Methylotenera* and *Stenotrophomonas*), accounting for 54% of these microbes. Genera from Proteobacteria represented 55% (*n*=26) and 50% (*n*=8) of the overabundant microbes in the foregut of fish with low and high metabolic rates, respectively. Other commonly overabundant taxa were from Actinobacteria (e.g. *Microbacterium* and *Friedmanniella*), Bacteroidetes (e.g. *Polaribacter* and *Chryseobacterium*) and Firmicutes (e.g. *Trichococcus* and *Bacillus*). Actinobacteria and Bacteroidetes each accounted for 14% (*n*=9) of the differentially abundant genera, with most genera more abundant in fish with low SMR (6 of 9 Actinobacteria and 7 of 9 Bacteroidetes genera). Firmicutes represented 8% (*n*=5) of the differentially abundant genera, with 4 of the 5 genera overabundant in fish with low SMR.

In the hindgut, 32 of the 55 differentially abundant genera were overabundant in fish from the low SMR group (Fig. 6B). As before, the most common differentially abundant genera belonged to Proteobacteria (e.g. *Loktanella* and *Brevundimonas*), accounting for 56% of these microbes. Proteobacteria represented 59% (*n*=19) and 52% (*n*=12) of the overabundant genera in the hindgut of fish with low and high metabolic rates, respectively. Similar to the foregut, other overabundant genera belonged to Actinobacteria (e.g. HGCL clade and *Streptomyces*), Bacteroidetes (e.g. *Ulvibacter*) and Firmicutes (e.g. *Lactococcus* and *Weisella*). Actinobacteria accounted for 16% (*n*=9) of the differentially abundant genera; however, unlike in the foregut, these genera were more abundant in fish with higher SMR (8 of 9 genera). Bacteroidetes and Firmicutes represented 13% (*n*=7) and 7% (*n*=4) of the differentially abundant genera, respectively, and were more commonly overabundant in fish with lower SMR (all 7 Bacteroidetes and 3 of 4 Firmicutes genera). Most other genera did not differ significantly between SMR groups, indicating that the observed compositional differences were driven by a subset of the community rather than a community-wide shift. These patterns suggest that variation in SMR is linked to changes in particular functional groups of bacteria rather than broad restructuring of the hindgut microbiota.

### Metabolic rate-linked variations in foregut microbiota correlate with *Rhodobacteraceae* abundance

**Fig. 7.**
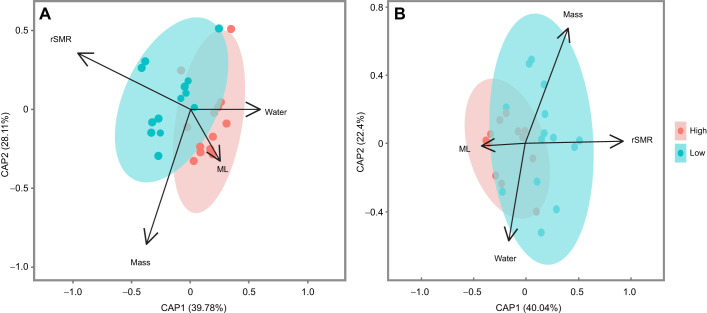
**Distance-based redundancy analysis (dbRDA) illustrating the drivers of differences in gut beta diversity between juvenile Atlantic salmon with low and high SMR.** Data are shown separately for the foregut (A) and hindgut (B). Blue and red points represent fish from low and high SMR groups, respectively. Arrows denote the magnitude and direction of the effects of explanatory variables. The total variance (as a percentage) explained by each axis is indicated. Mass (*P*=0.017) and rSMR (*P*=0.0073) were found to be significant drivers within the foregut. ML, microbial load.

The potential drivers of microbial community composition (as shown in Fig. 5) were also assessed using dbRDA to explore whether the variation seen within microbial communities between the low and high SMR groups was attributable to environmental variables. The dbRDA analysis revealed significant differences in the microbial community composition of the foregut, with the overall model explaining 22.27% of the observed variance (*P*=0.0012). Notably, fish mass and SMR contributed 6.26% and 6.90% of the variance, respectively, indicating their importance in shaping microbial communities (Fig. 7A). In contrast, the dbRDA model for the hindgut revealed no significant associations, suggesting that the examined variables had no influence on the microbial community structure (Fig. 7B).

In these dbRDA plots, samples positioned closer together represent communities with more similar microbial compositions, and the direction and length of each arrow indicate how strongly the corresponding variable influences the variation along each canonical axis.

Finally, Spearmen correlation coefficients were calculated to assess whether any metavariable (fish mass, fish length, DRER, growth efficiency, log-transformed average microbial load, fish water content and rSMR) correlated with any of the OTUs identified within the gastrointestinal samples. Samples from the foregut and hindgut of juvenile Atlantic salmon were assessed separately, but all fish from both the low and high SMR groups were analysed together, treating rSMR as a continuous variable to understand the relationships between the metavariables and OTUs. In both analyses, a FDR correction was applied before assessing significance.

**Fig. 8.**
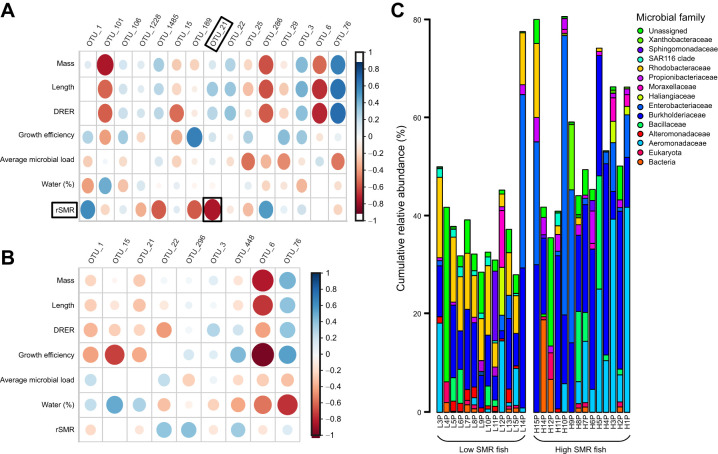
**Correlation plots showing relationships between metavariables and operational taxonomic units (OTUs) in the gut of juvenile Atlantic salmon.** Circle colour indicates the direction of the correlation (red, negative; blue, positive), and size reflects uncorrected *P*-values, with larger circles representing stronger correlations. Statistically significant correlations after false discovery rate (FDR) correction are marked with a bold black box. (C) Stacked bar plots depicting the taxonomic composition and cumulative abundance of microbial families in the foregut of fish with high and low metabolic SMR. Only microbial families with an abundance greater than 0.5% are shown for clearer visualisation.

Within the foregut of the juvenile Atlantic salmon, there was a significant negative correlation between rSMR and OTU 21 (*r*_14_=−0.81, *P*=0.017), which is a member of the Rhodobacteraceae family, belonging to the Proteobacteria phylum (Fig. 8A). Furthermore, an increased abundance of Rhodobacteraceae was found in the foregut of fish with a lower metabolic rate. This is illustrated in the stacked bar plots showing taxonomic composition at the family level (Fig. 8C). In contrast, within the hindgut, no significant correlations were found between any OTU and any metavariable after applying a FDR correction (Fig. 8B).

## DISCUSSION

In this study, we aimed to establish whether there is any link between gut microbial communities, digestive efficiency and metabolism in juvenile salmon. By analysing size-corrected metabolic rate and microbial composition, relationships were established between metabolic rate, growth efficiency, body water content (a proxy for fat content) and gut microbiota. We found that fish with a higher SMR appeared to be more efficient at extracting calories from their diet and had lower water content than those with lower SMR, indicating higher fat content and suggesting distinct energy allocation strategies. Larger fish were also apparently more efficient at recovering energy from their feed. Additionally, significant differences in gut microbial community composition were observed between fish of differing metabolic rate, and fish with higher SMR exhibited reduced microbial alpha diversity in the foregut. Beta diversity and differential abundance analysis highlighted variations in gut microbial taxa, with certain members of the Rhodobacteraceae family being more abundant in fish with low SMR. dbRDA further suggested that fish body mass and relative SMR influence gut microbial composition. Because our analyses focused on fish from the upper and lower ends of the SMR distribution, the results represent the extremes of metabolic phenotypes. This approach enhances contrast between groups but may limit generalisability to intermediate individuals. We note that VR is a proxy for SMR and, although widely used in salmonids, it lacks the precision of modern respirometry approaches.

### Impact of metabolic rate on growth efficiency and energy allocation

Juvenile brown trout can adjust their metabolic rates, lowering them during food scarcity and raising them when resources are plentiful (Auer et al., 2015a). This metabolic flexibility shows intraspecific variation (O'Connor et al., 2000), leading to different responses to environmental challenges. Regardless of this flexibility, some fish tend to have higher metabolic rates than others of the same size. Those with relatively higher metabolic rates often grow faster and secure better territories when food is abundant, but this advantage diminishes under adverse conditions (Reid et al., 2012). Understanding the links between fat levels, growth efficiency and metabolic rate is therefore essential for understanding the ecology of wild Atlantic salmon. While many studies have investigated the relationship between metabolic rate and body mass (Scheuffele et al., 2021; Segura-Hernández et al., 2024; Li et al., 2020), accounting for individual size enables a more focused examination of how size-corrected metabolic rate influences factors such as growth efficiency, energy harvest from nutrition and the composition of the gut microbial community. By controlling for body size, we were able to analyse the impact of size-corrected metabolic rate on growth efficiency and nutritional energy harvest. Our findings showed that fish with higher SMR grew more efficiently, incorporating a greater proportion of ingested energy into body growth (Fig. 3A). However, there was no correlation between metabolic rate and DRER (Fig. 2).

Although higher SMR fish grew more efficiently, incorporating a greater proportion of energy into growth, they did not retain more total energy overall, probably because of differences in body size or energy allocation strategy. This aligns with some studies in juvenile brown trout showing weak or negative correlations between metabolic rate and growth rate (Álvarez and Nicieza, 2005). However, other studies have reported positive associations under certain conditions, such as when food availability is high or predictable (Archer et al., 2021; Auer et al., 2015b), highlighting that the growth benefits of a high SMR are context dependent. The relationship between body mass and growth efficiency persisted even after accounting for metabolic rate.

Typically, SMR reflects the cost of maintaining energetically expensive tissues such as muscle (Auer et al., 2017; McKenzie et al., 2021). Contrary to this assumption, we found that individuals with higher SMR had higher fat levels, possibly because they could invest more energy in growth and fat storage under stable conditions with ample food. Fat levels in salmonids play a decisive role in life-history decisions such as sexual maturation, spawning and migration (Jonsson and Jonsson, 2005), directly affecting growth and metabolic rate. This suggests that under favourable feeding conditions, higher SMR fish may allocate energy towards fat storage, a strategy consistent with known salmonid energy accumulation patterns (Swift, 1955; Wootton et al., 2022).

Our results highlight that while the metabolic rate affects growth efficiency, understanding the role of gut microbiota in energy recovery is essential. Differences in gut microbial composition were evident between fish with low and high SMR. The increased microbial richness in the foregut of lower SMR fish is associated with differences in energy utilisation. Specifically, we observed a negative correlation between SMR and the abundance of members of the Rhodobacteraceae family, which were more common in the foregut of fish with low SMR. This is consistent with previous studies showing that certain gut bacteria are associated with enhanced digestion and nutrient absorption (Kokou et al., 2022; Bledsoe and Small, 2023). However, direct evidence of microbial influence on calorie recovery remains limited, indicating the need for further research to elucidate these relationships. Several microbial traits may help explain why higher foregut diversity is associated with more efficient energy use.

Communities with higher microbial richness may host a broader repertoire of carbohydrate-degrading enzymes that enable the breakdown of otherwise inaccessible dietary components (Wang et al., 2024; Barcan et al., 2024). Diverse taxa can also engage in cross-feeding interactions, where metabolites produced by one microbe serve as substrates for another, increasing overall nutrient extraction (Nandy et al., 2021). These microbial processes could help explain variation in caloric recovery even when total food intake is unchanged, offering a mechanism that complements the metabolic differences observed among individuals. Our research also suggest that, even under stable conditions, inherent differences in metabolic rate can lead to variation in growth among fish, reflecting the important of individual metabolic phenotypes (Huntingford and Adams, 2005). It is also notable that many taxa and functions did not differ significantly between metabolic groups. This is not unexpected, as the hindgut tends to host more stable core microbial members that vary less in response to host metabolic phenotype. In addition, differences in SMR may influence only particular functional groups rather than driving community-wide restructuring, which is consistent with the patterns observed in Fig. 6. The reduced sequencing sample size also limits power to detect subtle differences. Considering both significant and non-significant outcomes helps clarify that only a subset of the microbiota appears to be linked to variation in metabolic phenotype.

### Body composition and water content in relation to growth efficiency

Growth efficiency was negatively related to percentage water content (Fig. 3B). Fish with lower water content have proportionally fatter and less muscle (Berg and Bremset, 1998; Elliott, 1976a; Pyz-Łukasik et al., 2020). This indicates that fish assimilating more ingested energy were able to lay down more fat. Across all samples, a negative relationship between SMR and water content indicated that fish with higher SMR had more fat (Fig. 3C). This allocation of energy towards fat storage in higher SMR fish may be advantageous under stable conditions with ample resources. Some wild fish reduce feeding during winter to minimise energy expenditure (Bull et al., 1996), while others continue to feed despite insufficient intake to maintain energy reserves (Grade and Letcher, 2006). Seasonal changes significantly impact fat and water proportions in salmonids (Berg and Bremset, 1998; Finstad et al., 2009), with fat acting as a critical energy reserve. The link between fat content, growth efficiency and metabolic rate is particularly interesting because salmonids in the wild exhibit significant variation in body composition throughout their lifespan (Berg and Bremset, 1998; Elliott, 1976a; Swift, 1955; Iosilevskii et al., 2022).

These shifts in body composition reflect adaptations to environmental changes, which influence metabolic rate (O'Connor et al., 2000). Factors such as reduced food availability and lower temperatures often result in decreased somatic energy content over winter, followed by an increase during spring and summer months (Berg and Bremset, 1998). Maintaining energy stores is vital for survival, as energy deficiency is a major factor in winter mortality for juvenile Atlantic salmon (Finstad et al., 2011; Foddai et al., 2022). Thus, higher fat levels in high SMR fish may provide an energy buffer during periods of resource scarcity.

### Gut microbial diversity linked to energy utilisation and growth

This study explored the relationship between metabolic phenotype and gut microbiota in juvenile Atlantic salmon. Environmental water and biofilm samples were analysed only to provide environmental context and were not interpreted as part of the host–microbiome associations. While microbial load in faeces did not differ significantly between low and high SMR groups (Fig. S1), analysis showed that fish with lower SMR had higher microbial richness and Shannon diversity in the foregut (Table 2). This suggests that higher microbial diversity may contribute to energy utilisation. These differences represent correlations rather than a mechanistic effect of OTU richness on metabolic rate. In the hindgut, no significant relationships between metabolic rate and alpha diversity were found. However, growth efficiency increased with log-transformed average microbial load in the hindgut (Fig. S2), indicating that microbial load, even without significant diversity changes, may influence growth (Table S2).

Significant differences in microbial beta diversity were observed between low and high SMR groups in both the foregut and hindgut. This supported the hypothesis that variation in metabolic rate is associated with distinct microbial communities. Differential abundance analysis showed that Proteobacteria, Actinobacteria, Bacteroidetes and Firmicutes were present in varying proportions across metabolic groups. Previous research indicates that Tenericutes, Firmicutes, Bacteroidetes and Proteobacteria dominate the gastrointestinal tract of Atlantic salmon (Fogarty et al., 2019).

Microbial composition is known to vary across gut regions of fish, with Proteobacteria predominantly colonising the mucosa and both Proteobacteria and Firmicutes dominating the digesta (Gajardo et al., 2016; Jiao et al., 2023). Environmental changes, such as freshwater to saltwater transitions, can shift microbial prevalence (Rudi et al., 2018; Llewellyn et al., 2016), as can starvation (Xia et al., 2014). Our analysis found that Actinobacteria, Bacteroidetes and Firmicutes were more common in the foregut of low SMR fish, extending into the hindgut, except for Actinobacteria, which were higher in high SMR fish (Fig. 6). Such spatial variation within the gut highlights the nuanced interplay between microbial ecology and host physiology, shedding light on mechanisms that guide energy allocation and adaptation in salmonids.

Different microbial communities are known to have distinct functional profiles that impact the host in various ways. For example, anaerobic microbes in the gut produce short-chain fatty acids, which influence lipid, glucose and cholesterol metabolism in the host (Ray et al., 2012; den Besten et al., 2013). Specific taxa, such as *Pseudomonas* (a member of Proteobacteria), have been identified as recurrent members of fish gut microbiota, regulating host nutrient metabolism through gene expression (Nayak, 2010; Cheaib et al., 2021; Navarrete et al., 2009).

The dbRDA analysis demonstrated that foregut microbial composition is strongly shaped by host physiological traits, with body mass and metabolic rate profoundly influencing community structure (Fig. 7A). Correlation analysis highlighted a negative relationship between SMR and the Rhodobacteraceae family, with greater abundance in low SMR fish. In particular, *Ascidiaceihabitans* and *Octadecabacter* were prominent in low SMR fish (Fig. 6A,B), indicating that these taxa, and by extension, Rhodobactereacea more broadly, may be linked to the differential metabolic strategies observed in juvenile salmon Rhodobacteraceae (Fig. 8C). Rhodobacteraceae are known to play pivotal roles in nutrient cycling, including nitrogen assimilation and sulfur oxidation, processes that enhance environmental stability and host health (Rajeev and Cho, 2024). These processes can have substantial metabolic benefits, potentially lowering the energetic cost of homeostasis by improving nutrient utilisation and detoxification pathways.

### Implications and future directions

The correlation found between the Rhodobacteraceae family and metabolic rate is intriguing, but cannot reveal causality of any such relationship. Experimental methods, including microbial metabolite analysis and dietary interventions, could elucidate the functional roles of specific taxa (Lindsay et al., 2020). Diet influences gut microbiota composition (Green et al., 2013; Xia et al., 2014; Liu et al., 2022; Jin et al., 2022), and targeted use of probiotics or prebiotics may enhance energy recovery in aquaculture (Nayak, 2010; Llewellyn et al., 2014; Kazlauskaite et al., 2022; Abid et al., 2013).

Furthermore, understanding the impact of environmental and seasonal factors on gut microbiota is critical for assessing energy allocation and recovery. As our experiment highlighted these relationships in a stable laboratory environment, future work should examine these links across different environments, seasons and life stages, incorporating varied diets, body compositions and physiological states.

### Conclusions

Unveiling how metabolic phenotypes converge with gut microbial communities offers valuable insights into fish biology and energy management. Marked differences in gut microbial assemblies between high and low SMR groups suggests that fish metabolism and resident microbes may have co-evolved, enabling efficient energy utilisation and enhanced growth under diverse environmental conditions. Such cooperation potentially confers an evolutionary advantage by allowing fish to adapt to stressors through coordinated adjustments of both their internal metabolic processes and microbial partners.

## Supplementary Material

10.1242/jexbio.251523_sup1 Supplementary information

Dataset 1. Individual-level physiological and microbiome data for juvenile Atlantic salmon
