# Supplementary material for: Gut microbial composition varies with host metabolic phenotype in juvenile Atlantic salmon
Source: J Exp Biol. 2026 May 14;229(9):jeb251523. doi: 10.1242/jeb.251523 (PMC13245908; doi:10.1242/jeb.251523)
Supplement: Supplementary information [file jexbio-229-251523-s1.pdf]

Table S1. Summary of Equations Used in the Study

Equation Number	Equation	Purpose	Explanation (Brief)
Eq. 1	SMR = m(VR) + c	Converts ventilation rate (VR) into standard metabolic rate (SMR).	Uses a calibration model from Millidine et al. (2008) to translate opercular beats per minute into metabolic rate.
Eq. 2	$m = 0.2773 - [0.2350 \times \log_{10}(M)] - [0.01838T] + [0.05813T \times \log_{10}(M)] / 9$	Calculates the slope (m) of the VR–SMR relationship.	Adjusts the sensitivity of VR to SMR based on fish mass (M) and water temperature (T).
Eq. 3	$c = -3.4078 + 0.2958T + [2.1956 \times \log_{10}(M)] - [0.82057T \times \log_{10}(M)] + 0.5335M$	Calculates the intercept (c) of the VR–SMR relationship.	Determines baseline metabolic rate independent of ventilation effort, corrected for fish size and temperature.
Eq. 4	$M = 2.956 \times 10^{-5} (L)^{2.789}$	Predicts mass (M) from length (L) for ration calculations.	Provides a standardized estimate of body mass unaffected by temporary body condition, used to calculate feeding ration accurately.
Eq. 5	$SMR = 0.3544 \times M - 2.4856$ (R^2 = 0.70)	Regression of SMR against mass used to calculate relative SMR (rSMR).	Expected SMR for a fish of a given mass; residuals from this line classify fish into “high” or “low” metabolic phenotypes.

Supplementary Materials and Methods

Section 1: Residual Calculations for Energy and DRER

Residual Energy Content Calculation: To illustrate the relationship between faecal energy content ($kJ \cdot g^{-1}$) and fish mass, the expected energy content was calculated by controlling for DRER, log-transformed average microbial load, percentage water content, and rSMR:

$$Expected\ faecal\ energy\ content = (-0.34309 \times DRER) + (0.11825 \times microbial\ load) + (-0.06416 \times water\ content) + (-0.18841 \times rSMR) + 5.95731$$

Residuals were obtained by subtracting expected values from actual values and plotted against fish mass.

$\boldsymbol{Residual\ DRER\ Calculation}$: $The\ expected\ DRER\ was\ calculated\ as$: $Expected\ DRER = (-0.0247 \times scaled\ kJ\ consumed) + 4.1439$

Journal of Experimental Biology • Supplementary information

Residuals were plotted against fish mass to depict the relationship.

Section 2: Growth Efficiency and Water Content Residuals

Growth Efficiency Calculation: To accurately present the relationship between growth efficiency and rSMR, the expected scaled growth efficiency was calculated as:

$$Expected\ growth\ efficiency = (0.018 \times fish\ mass) + 0.1138$$

Residuals were derived by subtracting the expected growth efficiency from the actual values and plotted against rSMR (see Figure 3a in the main text).

Water Content Residuals: Expected water content was calculated as:

$$Expected\ water\ content\ (\%) = (-1.48 \times rSMR) + 73.021$$

Residuals were calculated by subtracting the expected values from the actual water content and plotted against growth efficiency (see Figure 3b in the main text).

Section 3: Microbial Diversity Metrics (Foregut)

Microbial Richness Calculation: The expected microbial richness was calculated as:

$$Expected\ microbial\ richness = (7.689 \times log - transformed\ microbial\ load) - 87.491$$

Residuals were obtained by subtracting expected values from actual microbial richness and plotted (see Figure 4a in the main text).

Shannon Effective Diversity Calculation: The expected Shannon effective was calculated as:

$$Expected\ Shannon\ effective = (9.493 \times percentage\ water\ content) - 636.03$$

Residuals were plotted against the log-transformed average microbial load (see Figure 4b in the main text).

Journal of Experimental Biology • Supplementary information

Growth Efficiency and Shannon Effective Residuals:

For analyzing the impact of Shannon effective diversity on growth efficiency, we computed the expected growth efficiency by excluding Shannon effective diversity from the model:

$$Expected\ growth\ efficiency = (0.0014180 \times microbial\ richness) + (0.0139520 \times microbial\ load) - (0.0915599 \times water\ content) + 6.7316$$

Residuals were derived and plotted against Shannon effective diversity (see Figure 4c in the main text).

Section 4: Microbial Load and Hindgut Analysis

Microbial Load: The log-transformed average microbial load (cfu·g^{-1}) in the faeces of juvenile Atlantic salmon showed no significant difference between the low and high rSMR groups (Welch's t-test, t23.6 = 0.18, P = 0.86; Figure S1). A linear model analysis found no significant effect of DRER, percentage water content, rSMR, or fish mass on microbial load (LM, all P > 0.23).

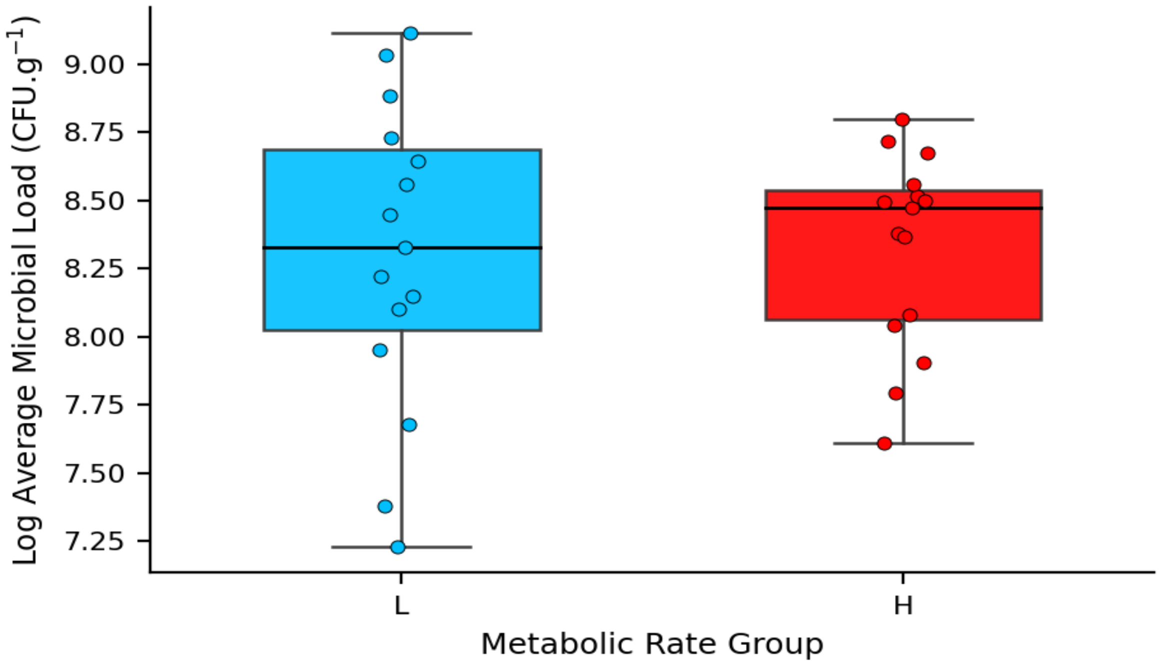

Fig. S1. The log-transformed average microbial load (cfu·g^{-1}) in the faeces of juvenile Atlantic salmon ('L': low; 'H': high relative SMR).

Journal of Experimental Biology • Supplementary information

Alpha Diversity Metrics in the Hindgut: Analysis showed no significant impact of any variable on hindgut microbial richness or Shannon effective diversity. The model assessing the influence of alpha diversity and physiological variables (fish mass, DRER, microbial load, and water content) on growth efficiency found that growth efficiency increased with microbial load (LM, F5, 11 = 42.72, P = 0.034) and decreased with water content (P < 0.001).

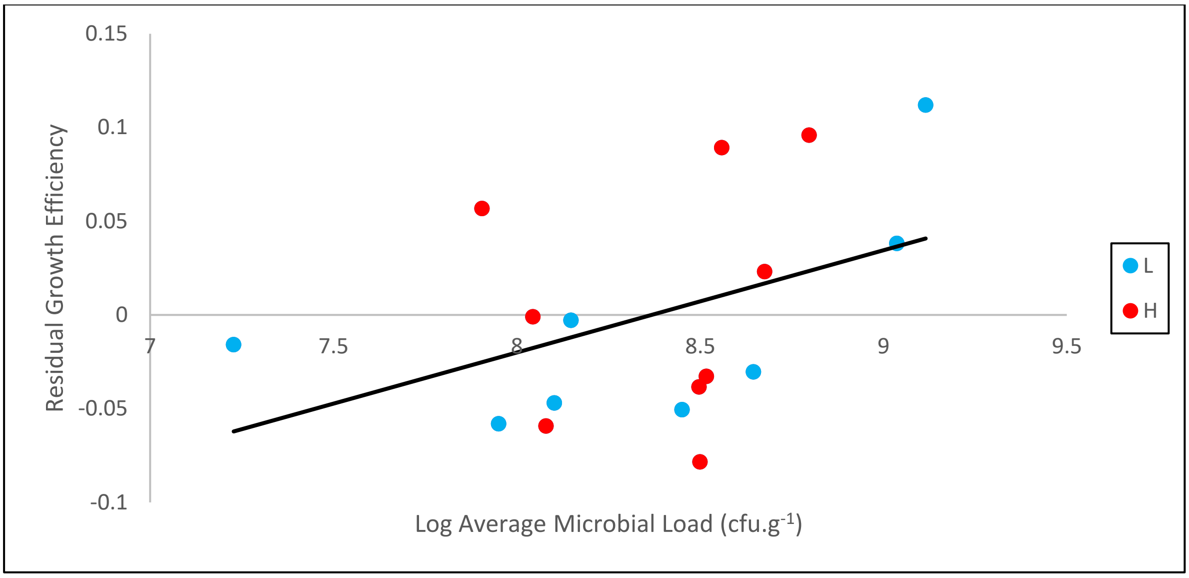

Fig. S2. The relationship between growth efficiency (standardized to 10g fish mass) and log-transformed microbial load in the hindgut, showing residual values after controlling for Shannon effective diversity, fish mass, DRER, and water content.

Within the hindgut of juvenile Atlantic salmon, no explanatory variable was found to impact either the microbial richness or the Shannon effective. As with the foregut samples, the impact of both alpha diversity metrics (this time pertaining to the hindgut) and the other metavariables on the growth efficiency (standardised to 10g fish mass) was also assessed. The final model had growth efficiency (standardised to 10 g fish mass) as the response variable and the Shannon effective, fish mass, daily relative energy retained (DRER), log-transformed average microbial load and percentage water content of the fish as explanatory variables (as summarised in Table S1). Growth efficiency was found to increase with the log-transformed average microbial load (LM, $F_{5, 11}$ = 42.72, P = 0.034) and, as with the foregut, decrease with percentage water content

Journal of Experimental Biology • Supplementary information

(P < 0.001). To present the relationship between growth efficiency (standardised to 10 g fish mass) and the log-transformed average microbial load, the expected growth efficiency (when accounting for the Shannon effective, fish mass, DRER and the fish percentage water content) was calculated as ((0.0011870 * Shannon effective) + (-0.0105962 * fish mass) + (0.0571968 * DRER) + (-0.0921459 * percentage water content) + 7.0513818). This expected growth efficiency was then subtracted from actual growth efficiency to give the residual values, which were then plotted against the log-transformed average microbial load.

Table S2. Linear Model Summary of Microbial Diversity and Salmon Growth. A summary of the linear model testing the relationships between microbial alpha diversity in the hindgut and salmon growth efficiency.
Efficiency

Response	Explanatory	t-value	P-value
	Shannon effective	2.03	0.068
	Mass	-1.47	0.17
Growth efficiency	Daily relative energy retained (DRER; kJ)	1.71	0.12
	Log-transformed average microbial load (cfu·g^{-1})	2.42	0.034
	Fish water content (%)	-13.60	<0.001

Section 5: Supplementary Data Description

Supplementary Data 1 (Excel file) contains the individual-level dataset used in this study. Each row corresponds to a single sample, identified by sample ID and barcode. The dataset includes experimental metadata (experiment, treatment group, sample type, origin), physiological measurements (mass, length, DRER, growth efficiency, water content, SMR, rSMR), and microbiological variables (average microbial load and alpha diversity metrics where available).

Journal of Experimental Biology • Supplementary information

Both foregut and hindgut samples are included, along with environmental samples where applicable. Missing values are indicated as NA. These data form the basis of all statistical analyses and figures presented in the manuscript. These data were used for all statistical analyses and figure generation presented in the manuscript.

Section 6: R codes

```
# ================================

# 1. LOAD LIBRARIES

# ================================

library(ggplot2)

library(dplyr)

# ================================

# 2. LOAD DATA

# ================================

metadata <- read.delim("metadata.tsv", header = TRUE)

alpha <- read.delim("exp2-alpha-diversity-fore.tab", header = TRUE)

# ================================

# 3. PREPARE MICROBIAL LOAD (FIG S1)

# ================================

df <- metadata %>%
```

Journal of Experimental Biology • Supplementary information

```
  filter(Treatment %in% c("low", "high"))

# Create Fish ID (remove P/H)

df$Fish_ID <- gsub("[PH]$", "", df$Sample)

# Convert to numeric

df$Average_Microbial_Load <- as.numeric(df$`Average Microbial Load`)

# Keep one value per fish

df <- df %>%

  arrange(Fish_ID, Sample_Type)

fish <- df %>%

  group_by(Fish_ID) %>%

  slice(1) %>%

  ungroup()

# Log transform

fish$log10_load <- log10(fish$Average_Microbial_Load)

# ================================

# 4. WELCH T-TEST (FIG S1)

# ================================

low <- fish %>% filter(Treatment == "low") %>% pull(log10_load)

high <- fish %>% filter(Treatment == "high") %>% pull(log10_load)

t_test <- t.test(low, high)
```

Journal of Experimental Biology • Supplementary information

```
print("Welch t-test (Figure S1)")

print(t_test)

# ================================

# 5. PLOT FIGURE S1

# ================================

ggplot(fish, aes(x = Treatment, y = log10_load, color = Treatment)) +

  geom_boxplot(outlier.shape = NA) +

  geom_jitter(width = 0.1, size = 2) +

  scale_color_manual(values = c("low" = "deepskyblue", "high" = "red")) +

  labs(

    x = "Metabolic Rate Group",

    y = "Log Average Microbial Load (CFU.g⁻¹)"

  ) +

  theme_classic() +

  theme(legend.position = "none")

ggsave("Figure_S1.png", width = 5, height = 4, dpi = 300)

# ================================

# 6. PREPARE RICHNESS (FIG 4a)

# ================================

colnames(alpha)[1] <- "Sample"
```

```
# Assign group

alpha$Group <- ifelse(grepl("^L", alpha$Sample), "low", "high")

# Merge microbial load

alpha$Fish_ID <- gsub("P$", "", alpha$Sample)

alpha <- merge(alpha, fish[, c("Fish_ID", "log10_load")], by = "Fish_ID", all.x = TRUE)

# ================================

# 7. CALCULATE RESIDUAL RICHNESS

# ================================

model <- lm(Richness ~ log10_load, data = alpha)

alpha$Residual_Richness <- resid(model)

# ================================

# 8. PLOT FIGURE 4a

# ================================

ggplot(alpha, aes(x = Group, y = Residual_Richness, color = Group)) +

  geom_boxplot(outlier.shape = NA) +

  geom_jitter(width = 0.1, size = 2) +

  scale_color_manual(values = c("low" = "deepskyblue", "high" = "red")) +

  labs(

    x = "Metabolic Rate Group",
```

Journal of Experimental Biology • Supplementary information

```
    y = "Residual Microbial Richness"
  ) +
  theme_classic() +
  theme(legend.position = "none")
ggsave("Figure_4a.png", width = 5, height = 4, dpi = 300)
# ===============================
# 1. SET WORKING DIRECTORY
# ===============================
setwd("C:/Users/Dee Jay/Desktop/Jobs_to_do/Papers/SMR_manuscript/05.12/for publishing/Last_version/RAW")
# ===============================
# 2. LOAD LIBRARIES
# ===============================
library(ggplot2)
library(dplyr)
# ===============================
# 3. LOAD DATA
# ===============================
# Alpha diversity (richness)
alpha <- read.delim("richness/exp2-alpha-diversity-fore.tab", header = TRUE)
```

Journal of Experimental Biology • Supplementary information

```
# Fix sample column name

colnames(alpha)[1] <- "Sample"

# Metadata (the big table you sent)

meta <- read.delim("metadata_file_name_here.tab", header = TRUE)

# ================================

# 4. KEEP ONLY FOREGUT SAMPLES

# ================================

meta_fore <- meta %>%

  filter(Sample_Type == "foregut") %>%

  select(Sample, Treatment, `Average Microbial Load`)

# Rename for simplicity

colnames(meta_fore) <- c("Sample", "Group", "MicrobialLoad")

# ================================

# 5. MERGE DATA

# ================================

df <- merge(alpha, meta_fore, by = "Sample")

# ================================

# 6. PREPARE VARIABLES

# ================================

# Convert to numeric
```

```
df$MicrobialLoad <- as.numeric(df$MicrobialLoad)

df$Richness <- as.numeric(df$Richness)

# Log transform

df$logLoad <- log10(df$MicrobialLoad)

# ===============================

# 7. CALCULATE RESIDUAL RICHNESS

# ==============================

model <- lm(Richness ~ logLoad, data = df)

df$ResidualRichness <- resid(model)

# ===============================

# 8. PLOT FIGURE 4a

# ===============================

ggplot(df, aes(x = Group, y = ResidualRichness, color = Group)) +

  # Boxplot

  geom_boxplot(outlier.shape = NA, alpha = 0.7) +

  # All data points

  geom_jitter(width = 0.1, size = 2, stroke = 0.3) +

  # Colors

  scale_color_manual(values = c("low" = "deepskyblue", "high" = "red")) +

  scale_fill_manual(values = c("low" = "deepskyblue", "high" = "red")) +
```

```
  # Labels
  labs(
    x = "Metabolic Rate Group",
    y = "Residual Microbial Richness"
  ) +
  # Theme (clean like paper)
  theme_classic() +
  theme(
    legend.position = "none",
    axis.text = element_text(size = 12),
    axis.title = element_text(size = 13)
  )
# ================================
# 9. SAVE FIGURE
# ================================
ggsave("Figure4a_residual.png", width = 5, height = 4, dpi = 300)
```

Dataset 1. Individual-level physiological and microbiome data for juvenile Atlantic salmon

Available for download at
https://journals.biologists.com/jeb/article-lookup/doi/10.1242/jeb.251523#supplementary-data

Journal of Experimental Biology • Supplementary information
